# Trends in socioeconomic inequality in mortality during childhood between 1993 and 2021 in India

**DOI:** 10.1136/bmjgh-2024-016386

**Published:** 2025-05-02

**Authors:** Anoop Jain, Akhil Kumar, Thomas W Pullum, Rockli Kim, Soumya Swaminathan, S V Subramanian

**Affiliations:** 1 Environmental Health, Boston University School of Public Health, Boston, Massachusetts, USA; 2 University of Toronto, Toronto, Ontario, Canada; 3 Professor Emeritus, University of Texas at Austin, Austin, Texas, USA; 4 Health Policy and Management, Korea University, Seoul, Korea; 5 MS Swaminathan Research Foundation, Chennai, Tamil Nadu, India; 6 Harvard Center for Population and Development Studies, Cambridge, Massachusetts, USA

**Keywords:** India, Child health, Health policy, Cross-sectional survey, Epidemiology

## Abstract

**Introduction:**

In India, most child deaths now occur within the first 28 days of birth. Trends in socioeconomic disparities in death during these early and late neonatal stages over the past few decades have been understudied. This paper elucidates these trends in early neonatal and late neonatal mortality by household wealth and maternal education. We also examined these trends for post neonatal and child mortality, thereby examining the risk of death by socioeconomic status from birth until 59 months.

**Methods:**

Using data from five rounds of India’s National Family Health Survey, we examined how the early neonatal, late neonatal, post neonatal and child mortality rates changed between 1993 and 2021 by household wealth and maternal education. We also examined how the absolute (difference in rates) and relative (ratio of rates) inequality between the highest and lowest socioeconomic groups changed for each outcome, and which children are on track to meet the Sustainable Development Goal targets.

**Results:**

Despite large absolute reductions in early neonatal, late neonatal, post neonatal and child mortality, India’s most vulnerable children remain at the highest risk of death as of 2021. Between 1993 and 2021, the absolute and relative socioeconomic inequality for early neonatal deaths increased. Now, most child deaths are among India’s most vulnerable children in terms of household wealth and maternal education, and these children are not on track to meet the Sustainable Development Goal targets for early neonatal and post neonatal mortality.

**Conclusions:**

Our study highlights persistent socioeconomic inequalities in child death, and that these inequalities exist regardless of mortality stage. More pro poor policies and interventions are required to close these gaps. Doing so is essential for India to meet global targets.

**WHAT IS ALREADY KNOWN ON THIS TOPIC**

- While the burden of child death in India has decreased over the past few decades, far less is known about trends in under-5 mortality throughout India over the past several decades when stratifying by socioeconomic status.

**WHAT THIS STUDY ADDS**

- This study examines trends in early neonatal, late neonatal, post neonatal and child mortality between 1993 and 2021 by household wealth and maternal education.

**HOW THIS STUDY MIGHT AFFECT RESEARCH, PRACTICE OR POLICY**

- Our study highlights persistent socioeconomic inequalities in child death, and that these inequalities exist regardless of mortality stage. More pro poor policies and interventions are required to close these gaps.

## Introduction

 The Sustainable Development Goals (SDGs), established in 2015, have set country-level targets for preventing neonatal mortality and under-5 mortality. Countries agreed to reduce the neonatal mortality rates (NMRs) to at least 12 deaths per 1000 live births by 2030 and the under-5 mortality rate to at least 25 deaths per 1000 live births by 2030.^1^ Recent trend analyses show that as of 2019, 132 countries had already achieved or were on track to achieve, the SDG for neonatal mortality.^2^ As of 2019, 142 countries had already achieved or were on track to achieve, the SDG for under-5 mortality.^2^

These trend analyses provide insights into national progress. But progress can vary within countries, too. For example, rates of under-5 mortality vary within countries by household wealth, as measured by asset indices, and parental education, two important markers of socioeconomic status (SES).^3^ The poorest children within low-income and middle-income countries (LMICs) are less likely to survive through the neonatal period than their wealthier counterparts.^4^ Evidence from 92 countries shows that after controlling for other indicators of SES, low maternal and paternal education are significant risk factors for child mortality.^5^ These findings underscore the importance of centring equity in strategies aimed at preventing child mortality.^6 7^

The burden of child death in India further illustrates the importance of applying an equity lens when studying trends in under-5 mortality. For instance, India’s child mortality rate (CMR) fell from 33.5 to 6.9 deaths per 1000 live births between 1993 and 2021.^8^ As a nation, India is now on track to meet the SDG target for reduced child mortality by 2030 thanks to this progress.^8^ However, as of 2021, several states are still not on track to meet this target. At the current rates of decline, states such as Bihar, Manipur and Uttarakhand will not achieve the SDG target for child mortality by 2030.^8^ These findings underscore the importance of geographically targeting services.

Far less is known about trends in under-5 mortality throughout India over the past several decades when stratifying by SES. Various studies show socioeconomic disparities in child death at specific time points in India.^9^,^12^ Studies that do show how these inequalities have persisted over time are older and thus do not contain data after 2017.^13^,^18^ Furthermore, conducting an updated trend analysis is important because the timing of child deaths has changed over the past three decades.^19^ In India, approximately 58% of child deaths now occur in the early neonatal (0–7 days) and late neonatal (8–28 days) periods, up from 42.5% in 1993.^8^ One study shows wealth and maternal-education-related inequalities in early neonatal deaths in India between 1990 and 2006.^20^ Another study shows that this trend extended to 2016.^21^ However, neither of these studies examined trends for the late-neonatal period and did not include more recent data. Examining age-specific mortality is important as the aetiology of under-5 child death varies by age.^22^ A review of previously published studies that examine trends in markers of under-5 death in India by household wealth and/or maternal education is presented in online supplemental table 1.

This study uses data from five rounds of India’s National Family Health Survey (NFHS) to examine trends in age-specific mortality by household wealth and maternal education in children younger than 5 between 1993 and 2021. Filling this gap is critical from an equity perspective as it will elucidate which children, as a function of SES, need to be prioritised. In doing so, we examine the risk of child death by household wealth and maternal education between birth and day 7 (early neonatal mortality), between day 8 and day 28 (late neonatal mortality), from day 29 to 11 months (post neonatal mortality) and from 12 months to 59 months (child mortality) for the entire period spanning from 1993 to 2021.

## Methods

### Data

The data for this study were from five rounds of India’s NFHS, each of which was led by the International Institute for Population Sciences and the Government of India’s Ministry of Health and Family Welfare. NFHS-1 was conducted in 1992–1993, NFHS-2 was conducted in 1998–1999, NFHS-3 was conducted in 2005–2006, NFHS-4 was conducted in 2015–2016 and NFHS-5 was conducted in 2019–2021. Each NFHS follows the data collection and reporting protocols outlined by the Demographic Health Surveys (DHSs) Programme. These protocols are standard across 90 different countries.^23^ Child mortality data from each round were nationally representative.

### Study population

Each round of the NFHS contains a birth history module in which enumerators ask women about their full birth history. Children who died in the 5 years preceding the survey were included in this study. The DHS provided a detailed guide on how to impute the date of birth or age at death for children for whom that information is missing,^24^ which we followed for the purposes of this analysis. All births and deaths had valid values for the mother’s education and the household’s wealth quintile for all five NFHS surveys.

### Outcomes

We included four outcomes in this study. These were the early NMR (ENMR), the late NMR (LNMR), the post NMR (PNMR) and the CMR. For each of these outcomes, the survey observes the sample number of deaths that occurred approximately 5 years preceding the date of the mother’s interview in each survey. The time frame of 5 years is approximate because the metric used for age of death is not consistently measured for all deaths. For instance, deaths are measured in days for children who died within the first month after birth. But, for children who died after one month but before their second birthday, their age of death is measured in months. Finally, age of death is measured in years for children who died between their second and fifth birthdays. For example, the death of a child who died at age 2 but was born 90 months before the interview can only be placed in the range of 24–35 months after birth, or 55–66 months before the survey. We follow standard DHS procedures for allocating ambiguous deaths and risk to the outcomes in the past 5 years.^25^

The ENMR is defined as the number of children who die within the first 7 days after birth per 1000 live births.^26^ The LNMR is defined as the number of children who die between days 8 and 28 after birth per 1000 live births.^26^ The PNMR is defined as the number of children who die between 29 days and 11 months after birth per 1000 live births. Finally, the CMR is defined as the number of children who die between 12 and 59 months after birth per 1000 live births.

We used the true cohort life table approach, as suggested by the DHS,^25^ to estimate the ENMR and LNMR because these values apply to children under the age of 12 months. For the PNMR value, we calculated the difference between infant mortality (the sum of the ENMR, LNMR and PNMR) and neonatal mortality (ENMR and LNMR). While neonatal mortality can be calculated using the real birth cohort, infant mortality must be ascertained using the synthetic cohort lifetable approach. This process, which has been described by the DHS,^25^ involves calculating the probability of survival for 0, 1–2, 3–5 and6–12 months. The probability of death is 1 minus the probability of survival from each period. The infant mortality rate is the product of these component death probabilities subtracted from 1 and then multiplied by 1000. The synthetic cohort approach was also used to calculate the CMR with the component age brackets of 12–23 months, 24–35 months, 36–47 months and 48–59 months.^25^ We note that the post neonatal, infant and CMRs are compound rates, based on calculations for subintervals of age. The denominators of the underlying rates include any exposure to the risk of death within the combination of age and time, discounted for deaths at an earlier age. The births that precede these observed deaths (or that provide exposure to risk even if the child survived) constitute the denominators. For example, a child who died before the first birthday is not at risk of dying after the first birthday. The births are not restricted to the 5 years preceding each survey period but may have occurred up to ten years earlier. For example, a child who died just a month before the fifth birthday, in the past 5 years, could have been born up to (but not including) 10 years before the survey. Table 1 gives the number of deaths for the respective age intervals, and these values are weighted to adjust for the sample design. NFHS surveys, like all DHS surveys, tend to oversample the relatively small sampling strata and undersample the relatively large strata. Weights with an average value of one compensate for the over and under sampling. The codes to calculate the mortality rates by wealth quintile and maternal education were adapted from a previous publication that examined the state variation in the same mortality rates in India.^8^

**Table 1:** Unweighted number (%) of mothers in the sample with any birth in the past 10 years by household wealth quintile and maternal education

	1993	1999	2006	2016	2021
Household wealth quintile	n (%)	n (%)	n (%)	n (%)	n (%)
Lowest	10,257 (17.3)	10,180 (18.0)	8,556 (15.4)	70,346 (23.7)	70,265 (24.8)
Low	10,550 (17.8)	10,216 (18.0)	9,400 (16.9)	67,190 (22.7)	64,469 (22.7)
Middle	11,633 (19.6)	11,328 (20.0)	11,129 (19.9)	59,563 (20.1)	55,861 (19.7)
High	13,449 (22.7)	12,667 (22.3)	12,590 (22.6)	52,599 (17.8)	50,221 (17.7)
Highest	13,415 (22.6)	12,337 (21.7)	14,056 (25.2)	46,519 (15.7)	42,715 (15.1)
Maternal education	n (%)	n (%)	n (%)	n (%)	n (%)
No schooling	34,397 (58.0)	29,063 (51.2)	22,177 (39.8)	95,538 (32.3)	67,215 (23.7)
1st–5th grade	8,242 (13.9)	8,952 (15.8)	7,934 (14.2)	41,707 (14.1)	37,064 (13.1)
6th–8th grade	6,420 (10.8)	6,932 (12.2)	8,265 (14.8)	50,445 (17.0)	50,190 (17.7)
9th–12th grade	7,701 (13.0)	8,970 (15.8)	12,522 (22.5)	80,151 (27.0)	91,939 (32.4)
Above 12th grade	2,544 (4.3)	2,811 (5.0)	4,833 (8.7)	28,376 (9.6)	37,123 (13.1)

.

### Statistical analysis

#### Rates by household wealth and maternal education

We estimated these values by household wealth quintiles which are derived using principal component analysis on several housing characteristics and amenities. Each household member is assigned a score, and each household member is ranked according to this score. Household wealth quintiles were then ascertained by dividing these scores into five equal categories.^27^ While the assets and amenities used to construct the wealth quintiles are largely the same across survey periods, there are slight differences. For example, mobile phones are included in estimating household in NFHS 5 from 2021 but are not included in NFHS 1 from 1993. A full description of assets and amenities used in each survey round can be found on the DHS programme website.^28^ This does not undermine the comparability between survey rounds.^29^

We also estimated these values by maternal education. Each round of the NFHS has the number of years of education completed. These years correspond to grades. We used this information to create the following education categories. These were (a) no schooling (less than 1 year of school), (b) between 1st and 5th grade (between 1 and 5 years of schooling), between 6th and 8th grade (between 6 and 8 years of schooling), between 9th and 12th grade (between 9 and 12 years of schooling) and finally above 12th grade (more than 12 years of schooling which corresponds to undergraduate or graduate coursework).

#### Absolute and relative inequality

We calculated the absolute and relative inequality for each survey year for all four outcomes. The absolute inequality was calculated by subtracting the rate estimate among the highest SES children from the rate estimate among the lowest SES children (separately for household wealth quintile and maternal education). The relative inequality was calculated by dividing the rate estimate among the lowest SES children by the rate estimate among the highest SES children (separately for household wealth quintile and maternal education).

#### Share of mortality burden by household wealth quintile and maternal education

We calculated the percentage of total deaths in the early neonatal, late neonatal, post neonatal and child mortality periods by household wealth quintile and maternal education category. This was done by dividing the number of deaths in each SES category by the total number of deaths in the period. We did this for 1993 and 2021 to compare how the share of mortality burden has shifted between SES groups over time.

#### Progress towards the SDGs

The SDGs set targets for the NMR and under-5 mortality rate (U5MR). We use these two targets to approximate targets for the four outcomes in this study using a previously published procedure.^8^ The U5MR target is 25 deaths per 1000 live births. Of these 25 deaths, 12 are covered by the SDG target for neonatal mortality, which is between birth and 28 days. This corresponds to the early neonatal and late neonatal periods in our study (0–7 days and 8–28 days). Thus, for these two outcomes, we approximate the targets as 7 deaths per 1000 and 5 deaths per 1000, respectively (these values add up to 12, the NMR target). The remaining 13 deaths per 1000 live births are during what we term the post neonatal period (29 days to 11 months) and the child mortality period (12 to 59 months). For these outcomes, we set targets of 8 deaths per 1000 and 5 deaths per 1000, respectively (these values add up to 13).

To estimate progress towards these approximate goals, we calculated the needed rate of change needed to achieve these targets for each outcome based on the rate in 2016. This was done by subtracting the rate in 2016 from the target value and dividing by 14 (the number of years between 2016 and 2030). We then calculated the actual rate of change based on the standardised annual change (SAC) for each outcome between 2016 and 2021. This was done by subtracting the 2016 value from the 2021 value and dividing by 5, the number of years between 2016 and 2021. We then compare the actual rate to the needed rate to assess which children, based on SES, are on track to meet the SDGs. Using the SAC to assess progress towards the SDGs has been used in previous work.^8 30^

### Patient and public involvement

Patients were not involved in this study.

## Results

### Sample characteristics

In 1993 there were 2113 early neonatal deaths, 880 late neonatal deaths, 1892 post neonatal deaths, and 1991 deaths during the child mortality period. In 1999, there were 1808 early neonatal deaths, 623 late neonatal deaths, 1403 post neonatal deaths, and 1646 deaths during the child mortality period. In 2006, there were 1708 early neonatal deaths, 490 late neonatal deaths, 1049 post neonatal deaths, and 1043 deaths during the child mortality period. In 2016, there were 6218 early neonatal deaths, 1223 late neonatal deaths, 2861 post neonatal deaths, and 2310 deaths during the child mortality period. Finally, in 2021, there were 4836 early neonatal deaths, 979 late neonatal deaths, 2349 post neonatal deaths, and 1628 deaths during the child mortality period.

table 1

There was a shift in the household wealth and educational status of the mothers who had any birth in the past 10 years. In 1993, approximately 17.3% of mothers belonged to households in the lowest wealth quintile, while 22.6% of mothers were in the highest wealth quintile. By 2021, approximately 24.8% of mothers were in the lowest wealth quintile, while 15.1% were in the highest wealth quintile. For maternal education, approximately 58.0% of mothers had no schooling in 1993 while only 4.3% had received an education above the twelfth grade. By 2021, 23.7% of mothers had no schooling, while 13.1% had received above a 12th grade education.

These results are presented in table 1. We note that 185 women (<1% of sample) in 1993, 26 women (<1% of sample) in 1999, and 4 women in 2006 (<1% of sample) who had any birth in the past 10 years had missing education data. These women and their birth data were excluded from our analysis.

### Overall trends in indicators of child mortality between 1993 and 2021

Overall, the ENMR decreased for every wealth quintile and maternal education category between 1993 and 2021. The largest absolute declines in ENMR between 1993 and 2021 occurred for children in households that were in the low wealth quintile and for children with mothers with no schooling. As of 2021, the ENMR among the lowest wealth quintile households was 27.81 (95% CI 27.25 to 28.39), higher than the rate for the highest wealth quintile children in 1993. The ENMR was 27.52 (95% CI 26.92 to 28.13) among children with mothers with no schooling, higher than the rate for children with mothers who had above a 12th grade education in 1993. Additionally, there is a continued decline in the ENMR for all wealth quintiles or maternal education categories between 2016 and 2021.

The LNMR decreased for every wealth quintile and maternal education category between 1993 and 2021. The largest absolute decline in LNMR between 1993 and 2021 occurred for children in households in the low wealth quintile. This was also true for children with mothers with no schooling. As of 2021, the LNMR among the lowest wealth quintile households was 5.33 (95% CI 5.09 to 5.58), higher than the rate for the highest wealth quintile children. The LNMR was 5.66 (95% CI 5.39 to 5.94) among children with mothers with no schooling, higher than the rate for children with mothers who had above a 12th grade education in 1993. The LNMR also experienced a continued decline in all wealth quintiles and most maternal education categories between 2016 and 2021, except among mothers with 1st–5th grade education, which experienced an increase.

The PNMR decreased for every wealth quintile and maternal education category between 1993 and 2021. The largest absolute decline in PNMR between 1993 and 2021 occurred for children in households in the lowest wealth quintile and for children with mothers with no schooling. As of 2021, the PNMR among the lowest wealth quintile households was 14.81 (95% CI 14.80 to 14.81), higher than the rate for the highest wealth quintile children. As of 2021, the PNMR among children with mothers with no schooling was 15.85 (95% CI 15.85 to 15.85). This was higher than the PNMR among children with mothers who had above a 12th grade education in 1993. The PNMR increased between 2016 and 2021 for children living in households with the second highest wealth quintile and among children with mothers having a 1st–5th grade education, 6th–8th grade education and above a 12th grade education.

The CMR decreased for every wealth quintile and maternal education category between 1993 and 2021. The largest absolute decline in CMR between 1993 and 2021 occurred for children in households in the second lowest wealth quintile and for children with mothers with no schooling. As of 2021, the CMR among the lowest wealth quintile households was 11.60 (95% CI 10.55 to 12.65), higher than the rate for the highest wealth quintile children in 1993. The CMR was 11.84 (95% CI 10.76 to 12.92) among children with mothers with no schooling, higher than the rate for children with mothers who had above a 12th grade education in 1993. The CMR declined between 2016 and 2021 in all but one wealth quintile and maternal education category, which were the highest wealth quintile and mothers with above a 12th grade education.

**Figure 1:**
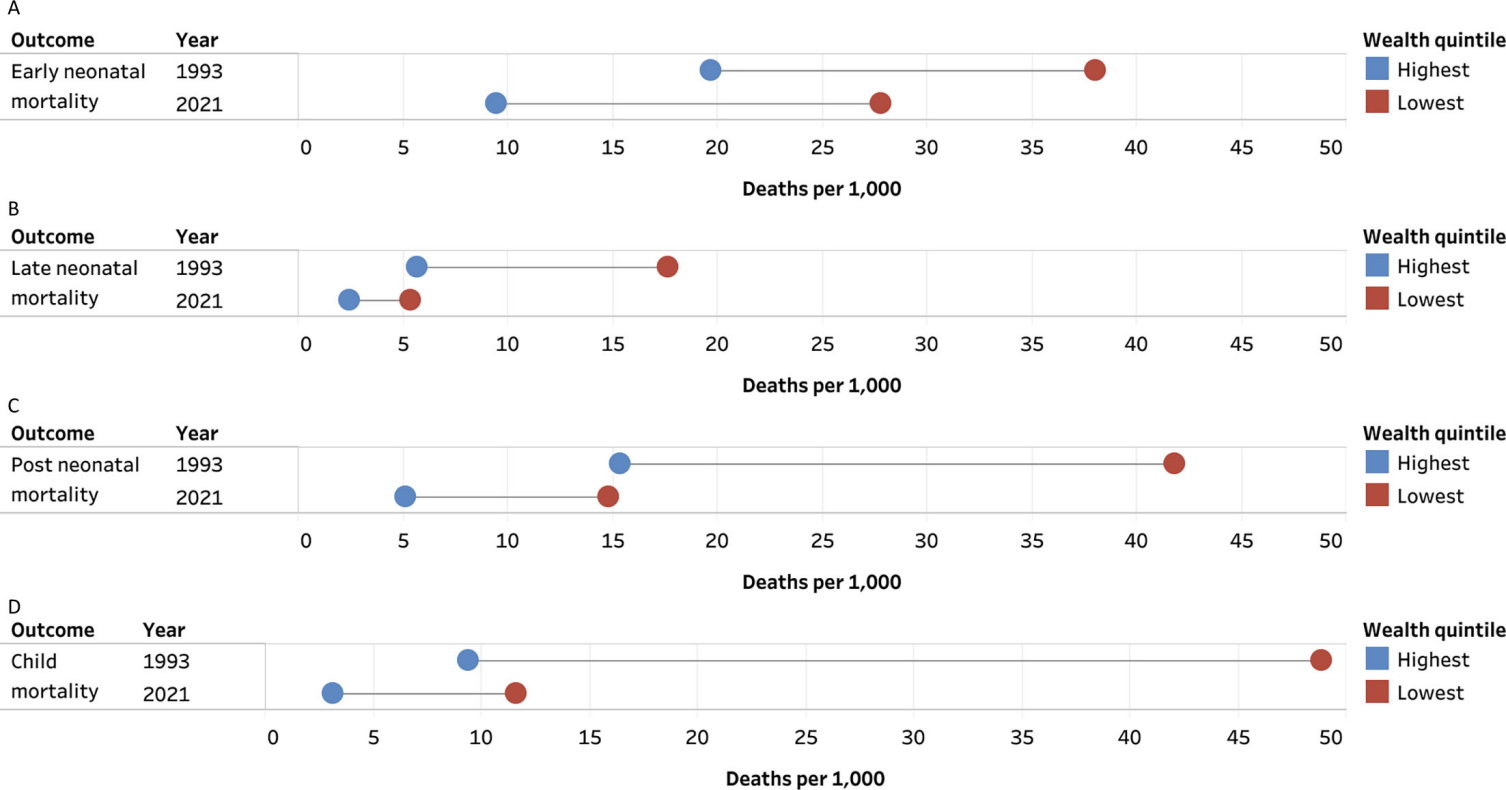
Deaths per 1000 live births by highest and lowest household wealth quintile in 1993 and 2021. (**A**) Early neonatal (0–7 days), (**B**) late neonatal (8–28 days), (**C**) post neonatal (1–11 months) and (**D**) child (12–59 months).

These results are presented in figures1  2 and online supplemental tables 2 and 3.

**Figure 2:**
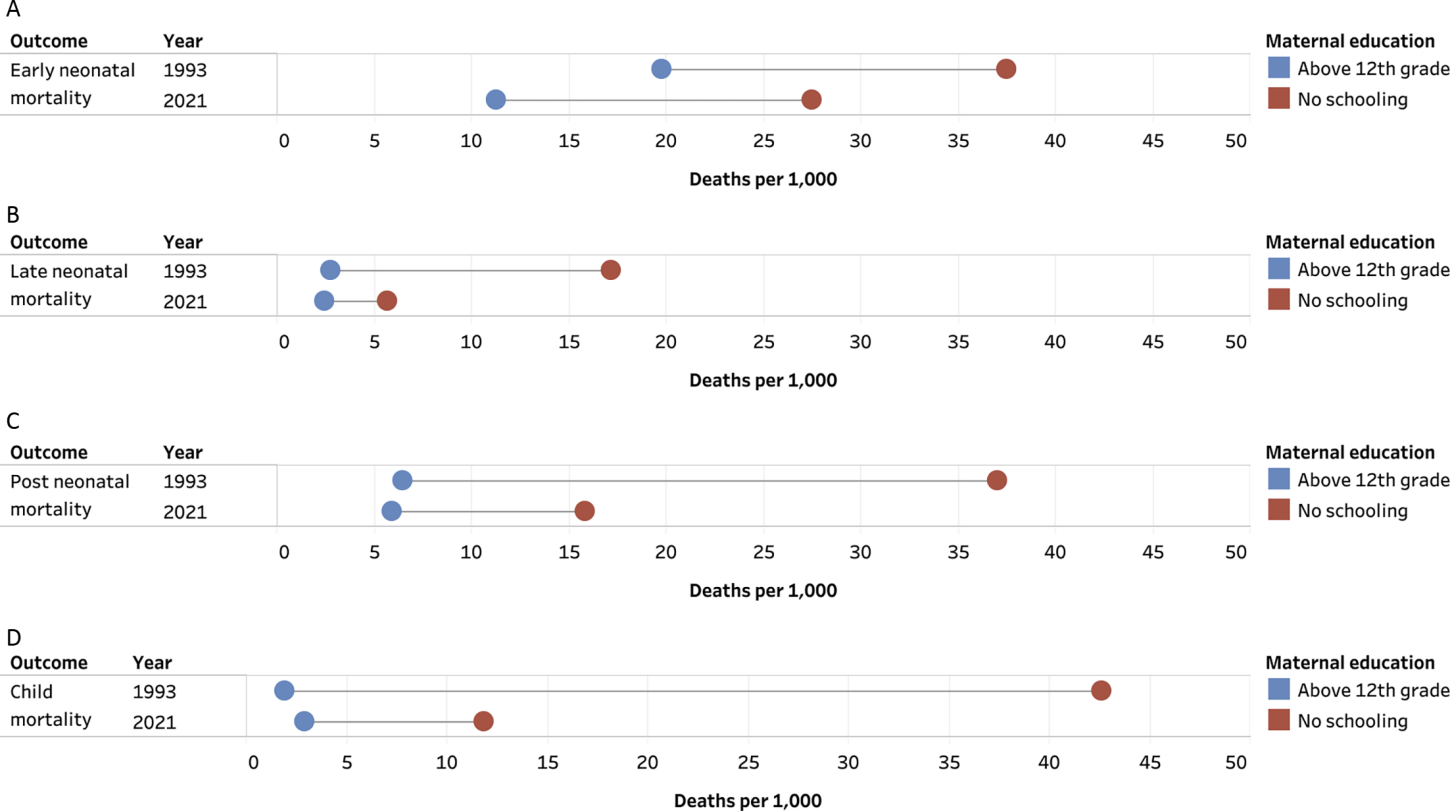
Deaths per 1000 live births by maternal education in 1993 and 2021. (**A**) Early neonatal (0–7 days), (**B**) late neonatal (8–28 days), (**C**) post neonatal (1–11 months) and (**D**) child (12–59 months).

### Changes in absolute and relative inequality between 1993 and 2021

The absolute inequality in ENMR between the lowest and highest wealth quintile households increased from 18.3 in 1993 to 18.4 in 2021, and the relative inequality between the lowest and highest wealth quintile households increased from 1.9 to 2.9 over that same period. The absolute inequality declined from 17.8 to 16.2 between mothers with no schooling and above a 12th grade education between 1993 and 2021. However, the relative inequality between these two groups increased from 1.9 to 2.4 over this period.

The absolute inequality in LNMR between the lowest and highest wealth quintile households decreased from 12.0 in 1993 to 2.9 in 2021. Similarly, the relative inequality in LNMR between the lowest and highest wealth quintile households decreased from 3.1 in 1993 to 2.2 in 2021. The absolute inequality declined from 14.4 to 3.2 between mothers with no schooling and above a 12th grade education between 1993 and 2021. The relative inequality between these two groups decreased from 6.2 to 2.3 over this period.

Between 1993 and 2021, the absolute inequality in PNMR decreased from 26.5 to 9.7 between the lowest and highest wealth quintile households. The relative inequality increased slightly from 2.7 to 2.9 over the same period between these wealth groups. The absolute inequality declined from 30.6 to 9.9 between mothers with no schooling and above a 12th grade education between 1993 and 2021. The relative inequality between these two groups decreased from 5.8 to 2.7 over this period.

Between 1993 and 2021, the absolute inequality in CMR decreased from 39.5 to 8.4 between the lowest and highest wealth quintiles households. The relative inequality decreased from 5.2 to 3.7 over the same period between these wealth groups. The absolute inequality declined from 40.7 to 9.0 between mothers with no schooling and above a 12th grade education between 1993 and 2021. The relative inequality between these two groups decreased from 22.2 to 4.1 over this period.

These results are presented in table 2.

**Table 2:** Absolute and relative inequality between the highest and lowest socioeconomic groups for the early neonatal, late neonatal, post neonatal and child mortality rates by survey year

	Early neonatal		Late neonatal		Post neonatal		Child	
	Absolute	Relative	Absolute	Relative	Absolute	Relative	Absolute	Relative
Household wealth quintile								
2021	18.4	2.9	2.9	2.2	9.7	2.9	8.4	3.7
2016	21.7	2.9	4.0	2.4	10.9	3.0	13.5	5.8
2006	17.8	2.0	7.8	3.6	15.7	3.1	27.5	6.8
1999	16.8	1.8	12.0	4.1	26.9	3.4	40.8	6.1
1993	18.3	1.9	12.0	3.1	26.5	2.7	39.5	5.2
Maternal education								
2021	16.2	2.4	3.2	2.3	9.9	2.7	9.0	4.1
2016	17.2	2.3	3.4	2.3	11.8	3.4	12.5	5.8
2006	20.0	2.4	8.9	7.7	20.1	5.0	23.1	7.1
1999	16.3	1.8	11.2	5.2	26.5	5.7	35.3	9.2
1993	17.8	1.9	14.4	6.2	30.6	5.8	40.7	22.2

The absolute inequality is a percentage point difference between the rate for the highest socioeconomic group and the rate for the lowest socioeconomic group for each outcome in a survey year. The relative inequality is the rate for the lowest socioeconomic group divided by the rate for the highest socioeconomic group for each outcome in a survey year.

### Share of deaths by SES

The burden of early neonatal, late neonatal, post neonatal and child deaths shifted between 1993 and 2021 such that in 2021, most deaths were among children in the two lowest wealth quintile households.

**Figure 3:**
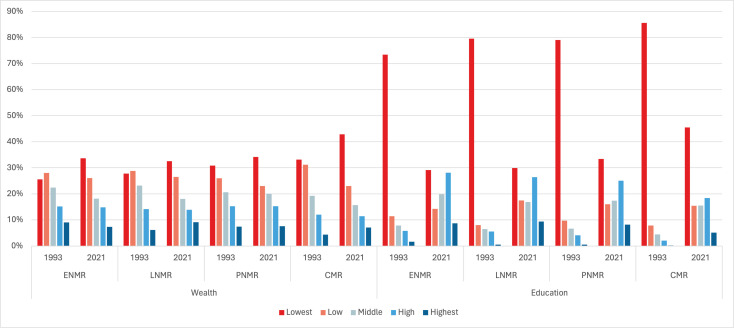
Share of deaths by household wealth quintile and maternal education for the early neonatal, late neonatal, post neonatal and child mortality periods in 1993 and 2021. Note: Lowest, low, middle, high and highest refer to wealth quintiles. For education, these refer to (a) no schooling, (**b**) 1st–5th grade, (**c**) 6th–8th grade, (**d**) 9th–12th grade and (e) above 12th grade. CMR, child mortality rate; ENMR, early neonatal mortality rate; LNMR, late neonatal mortality rate; PNMR, post neonatal mortality rate.

These results are presented in figure 3.

As of 2021, a lower share of deaths occurred among mothers with no schooling than in 1993. Despite this reduction, the largest share of deaths for each mortality period was still among mothers with no schooling as of 2021. There was an increase in the share of deaths among mothers with above a 12th grade education between 1993 and 2021.

These results are presented in figure 3.

### Progress towards the SDGs

The current rate of decline in ENMR is not sufficient for India to achieve the SDG target of 7 deaths per 1000 regardless of household wealth quintile or maternal education. The only exception to this is for the highest wealth quintile households, which had an actual SAC value of −0.42. For LNMR, at the current rate of decline, all children, regardless of household wealth or maternal education, either will, or already have, achieved the SDG target. The only exception for this is children with mothers with between a 1st and 5th grade education who currently have a positive SAC value of 0.09. For PNMR, with the exception of highest wealth quintile households and children with mothers with a 9th to 12th grade education or above a 12th grade education, the rate of decline is not sufficient to achieve the SDG target in 2030. Furthermore, the PNMR for children with mothers having above a 12th grade education increased between 2016 and 2021 indicating a future where it will no longer be meeting the SDG target soon after 2030. For CMR, at the current rate of decline, all children, regardless of household wealth or maternal education, either will or already have, achieved the SDG target. The only exception for this is children with mothers with no schooling who had an SAC value of −0.65. However, once again the CMR for children in the highest wealth quintile households and those with mothers having above a 12th grade education increased between 2016 and 2021 which indicates that this subpopulation may no longer meet the SDG target in the future.

These results are presented in online supplemental figures 1 and 2.

## Discussion

This study had four salient findings. First, the largest reductions in the ENMR, LNMR, PNMR and CMR were among children in the lowest SES groups. But the ENMR and CMR among the lowest wealth quintile households in 2021 were higher than the ENMR and CMR among the highest wealth quintile households in 1993. Similarly, the ENMR, LNMR, PNMR and CMR among children with mothers with no education in 2021 were higher than the ENMR, LNMR, PNMR and CMR among children with mothers who had above a 12th grade education in 1993. Second, while the absolute inequality and relative inequality declined between 1993 and 2021 for most outcomes, the absolute and relative wealth quintile inequality increased for ENMR, as did the relative education group inequality. The relative wealth quintile inequality increased slightly between 1993 and 2021 for PNMR as well. Third, a greater share of deaths at each period are now occurring among the lowest wealth quintile households in 2021 compared with 1993. While a smaller share of deaths occurred among children whose mothers have no schooling in 2021 compared with 1993, the largest share of deaths for each mortality period was still among mothers with no schooling as of 2021. Fourth, the SDG targets for ENMR and PNMR are out of reach for India’s most vulnerable children.

There are three possible data limitations to this study. First, there is a potential for recall bias as mothers are asked to self-report all births in the past 10 years and deaths in the past 5 years. However, previous work shows that recall bias has largely not impacted the estimation of under-5 mortality in studies that use DHS data.^31^ Second, education was also self-reported and could also be impacted by bias. Third, when evaluating which children are on track to meet the SDG targets, we assumed that the rate of change between 2016 and 2021 for the four outcomes would stay constant until 2030. However, it is possible that these rates could change, thereby altering SDG target projections.

Our results show large reductions in India’s ENMR, PNMR, LNMR and CMR among India’s most vulnerable children between 1993 and 2021. There are several possible explanations for these declines. India’s government has promoted antenatal care through programmes such as the Pradhan Mantri Surakshit Matritva Abhiyan, which provide free antenatal care to all pregnant women on the ninth of every month throughout the country. Similarly, India’s government has promoted institutional deliveries through programmes such as the Janani Suraksha Yojana which provide cash transfers, paying attention to India’s poorest women.^32^ Delivering in health facilities, or in the presence of skilled birth attendants, is another critical determinant of child survival.^33^,^37^ Postnatal care received by women can also help prevent child death.^38^,^40^ The Pradhan Mantri Matru Vandana Yojana centres the needs of India’s most socioeconomically disadvantaged mothers by providing them with cash to make up for lost wages so that they can receive care and take the adequate amounts of rest after birth.^41^ Improved mobile phone connectivity and road networks have also helped Indian women across the SES spectrum access care, and this could also help explain why India’s ENMR, PNRM, LNMR and CMR declined.^42^

But while India has made progress in addressing some of the determinants of child death, our results make clear that as of 2021, social and economic advantage has afforded some of India’s children a head start in the race towards better survival outcomes while others remain far off pace. The ENMR, PNMR and CMR among India’s least advantaged children in 2021 are higher than they were for India’s most advantaged children in 1993. The risk of death, at any point between birth and 5 years, is still several times greater among India’s least socioeconomically advantaged children than the highest and worsening for ENMR. And now, most of India’s child deaths are clustered among the most socioeconomically disadvantaged.

Our results are also consistent with findings from previous work showing that most child deaths occur within the first 28 days of life.^8^ We show that the bulk of these deaths occur among India’s lowest SES children. It is possible that this is being driven in part by congenital conditions, which remain extremely prevalent in India.^43^ But preventing early and late neonatal death among India’s lowest SES children will require concerted efforts to reduce socioeconomic inequality, a major driver of child mortality.^6^ We show that more women than ever before have attained higher levels of education throughout India. Yet wages in India, particularly among farmers and manual labourers, are stagnating.^44^ Poverty is also a root cause of low maternal body mass index,^45 46^ a predictor of low birth weight,^47^,^49^ a key determinant of early neonatal mortality.^8^ Maternal mortality is also highest in India’s poorest states,^50^ and it is possible this also contributes to high ENMR and PNMR among India’s lowest SES children. While most deaths occur in the first 28 days, India’s lowest SES children were also at the highest risk of death during the PNMR and CMR periods between 1 and 59 months. Poverty is a significant predictor of growth failure,^51^ a major determinant of child death in India at these later ages.^52^ It is also possible that these post neonatal and child deaths are occurring due to chronic food deprivation. Recent evidence from India shows that 30% of children between the ages of 6–12 months had not consumed any food in the past 24 hours.^53^ Similarly, many Indian children above the age of 12 are exclusively breastfed. This could deprive them of essential nutrients, thereby increasing their chance of death.^54^ Many of the government programmes noted above are trying to address these determinants. But in India, as is the case in other LMICs, care coverage remains the lowest for the poorest women.^55^ Furthermore, utilisation and quality of obstetric care is often lowest among the least socioeconomically advantaged women.^56^,^63^

## Supplementary material

10.1136/bmjgh-2024-016386 online supplemental file 1

10.1136/bmjgh-2024-016386 online supplemental file 2

## Data Availability

Data are available in a public, open access repository.
