# Supplementary material for: Trends in socioeconomic inequality in mortality during childhood between 1993 and 2021 in India
Source: BMJ Glob Health. 2025 May 2;10(5):e016386. doi: 10.1136/bmjgh-2024-016386 (PMC12049899; doi:10.1136/bmjgh-2024-016386)
Supplement: online supplemental file 1 [file bmjgh-10-5-s001.pdf]

Supplemental online content

Jain A, Kumar A, Pullum TW, et al. Trends in socioeconomic inequality in mortality during childhood between 1993 and 2021 in India. BMJ Glob Health 2025;0:e016386. doi:10.1136/ bmjgh-2024-016386

Table of Contents

Table/Figure	Title	Page
eTable1	Review of previously published studies that examine trends in markers of under-5 death in India by household wealth and/or maternal education	2
eTable2	Deaths per 1,000 live births [95% confidence interval] for early neonatal mortality, late neonatal mortality, post neonatal mortality, and child mortality by household wealth quintile in 1993, 1999, 2006, 2016, and 2021	3
eTable3	Deaths per 1,000 live births [95% confidence interval] for early neonatal mortality, late neonatal mortality, post neonatal mortality, and child mortality by household wealth quintile in 1993, 1999, 2006, 2016, and 2021	4
efigure 1	Standardized percent change for early neonatal, late neonatal, post neonatal, and child mortality by household wealth quintile and maternal education between 1993-1999, 1999-2006, 2006-2016, and 2016-2021	5
efigure2	Progress towards Sustainable Development Goal targets for ENMR, LNMR, PNMR, and CMR by household wealth quintile and maternal education as of 2021	6

This supplemental material has been provided by the authors to give readers additional information about their work.

Supplementary table 1: Review of previously published studies that examine trends in markers of under-5 death in India by household wealth and/or maternal education

Title	Marker of SES	Time range	Under-5 death measure	Data source
Is economic inequality in infant mortality higher in urban than in rural India?	Household wealth	1993, 1999, 2006	IMR	NFHS 1, 2, & 3
Household wealth and child health in India	Household wealth	1993, 1999, 2006	IMR, CMR, U5MR	NFHS 1, 2, & 3
Infant and child mortality in India: trends in inequalities across economic groups	Household wealth	1993, 1999, 2006	IMR, CMR	NFHS 1, 2, & 3
Socioeconomic and gender inequalities in neonatal, post neonatal, and child mortality in India: A repeated cross-sectional study, 2006-2016	Household wealth	2006-2016	NMR, PNMR, CMR	NFHS 3 & 4
Mind the gap: Temporal trends in inequalities in infant and child mortality in India (1992-2016)	Household wealth & maternal education	1993, 1999, 2006, 2016	IMR, CMR	NFHS 1, 2, 3, & 4
Infant and child mortality in India in the last two decades: A geospatial analysis	Household wealth & maternal education	1993, 1999, 2004	IMR, CMR	NFHS 1 & 2, and DLHS 2002-2004
Early neonatal mortality in India, 1990-2006	Household wealth & maternal education	1993, 1999, 2006	ENMR	NFHS 1, 2, & 3
Comparing socioeconomic inequalities between early neonatal mortality and facility delivery: Cross-sectional data from 72 low-and-middle-income countries	Household wealth & maternal education	1993, 1999, 2006, 2016	ENMR	NFHS 1, 2, 3, & 4

Supplementary table 2: Deaths per 1,000 live births [95% confidence interval] for early neonatal mortality, late neonatal mortality, post neonatal mortality, and child mortality by household wealth quintile in 1993, 1999, 2006, 2016, and 2021

	Lowest	Low	Middle	High	Highest
**Early neonatal mortality**					
**1993**	38.02 [36.62-39.47]	42.31 [40.84-43.83]	36 [34.65-37.4]	25.92 [24.8-27.08]	19.66 [18.55-20.84]
**1999**	37.75 [36.43-39.12]	33.4 [32.13-34.72]	32.55 [31.25-33.91]	27.88 [26.67-29.14]	20.95 [19.72-22.25]
**2006**	36.45 [34.98-37.98]	32.23 [30.78-33.75]	29.3 [27.86-30.81]	25.55 [24.2-26.97]	18.68 [17.43-20.01]
**2016**	33.22 [32.65-33.81]	28.39 [27.8-28.98]	23.17 [22.6-23.77]	17.96 [17.38-18.57]	11.55 [11.09-12.02]
**2021**	27.81 [27.25-28.39]	24.29 [23.73-24.86]	18.93 [18.37-19.5]	16.39 [15.8-16.99]	9.43 [9.01-9.87]
**Late neonatal mortality**					
**1993**	17.6 [16.68-18.56]	18.13 [17.19-19.12]	15.92 [15.02-16.88]	10.18 [9.49-10.9]	5.65 [5.1-6.25]
**1999**	15.89 [15.06-16.76]	12.78 [12-13.62]	10.46 [9.75-11.22]	7.3 [6.69-7.97]	3.88 [3.4-4.42]
**2006**	10.81 [10.03-11.65]	10.77 [9.95-11.66]	9.36 [8.54-10.26]	5.57 [4.98-6.24]	3.03 [2.57-3.57]
**2016**	6.76 [6.51-7.02]	5.23 [4.99-5.48]	4.45 [4.2-4.73]	3.34 [3.05-3.65]	2.8 [2.49-3.15]
**2021**	5.33 [5.09-5.58]	4.95 [4.69-5.21]	3.8 [3.54-4.08]	3.06 [2.84-3.31]	2.42 [2.11-2.79]
**Post neonatal mortality**					
**1993**	41.79 [41.79-41.8]	36.29 [36.28-36.29]	30.92 [30.91-30.92]	24.46 [24.46-24.46]	15.32 [15.31-15.32]
**1999**	38.08 [38.08-38.09]	28.64 [28.63-28.64]	24.87 [24.87-24.88]	16.1 [16.1-16.11]	11.18 [11.18-11.18]
**2006**	23.15 [23.15-23.16]	25.53 [25.52-25.53]	19.63 [19.63-19.64]	12.88 [12.87-12.88]	7.48 [7.48-7.49]
**2016**	16.33 [16.33-16.33]	13.55 [13.55-13.56]	11.52 [11.52-11.52]	8.33 [8.33-8.33]	5.47 [5.47-5.48]
**2021**	14.81 [14.8-14.81]	11.7 [11.7-11.7]	10.92 [10.92-10.92]	9.01 [9.01-9.01]	5.15 [5.15-5.15]
**Child mortality**					
**1993**	48.87 [44.46-53.27]	47.76 [43.27-52.24]	31.52 [27.77-35.27]	20.74 [17.8-23.68]	9.37 [7.19-11.56]
**1999**	48.81 [44.71-52.9]	37.2 [33.42-40.98]	24.87 [21.65-28.09]	13.98 [11.51-16.45]	8 [5.87-10.12]
**2006**	32.31 [28.53-36.09]	22.64 [19.1-26.18]	14.4 [11.45-17.35]	7.5 [5.41-9.58]	4.77 [2.94-6.6]
**2016**	16.29 [15.22-17.35]	10.64 [9.54-11.74]	7.29 [6.01-8.56]	5.43 [4.42-6.43]	2.8 [2.05-3.56]
**2021**	11.6 [10.55-12.65]	7.34 [6.43-8.25]	5.63 [4.64-6.63]	4.33 [3.38-5.29]	3.16 [2.34-3.98]

Supplementary table 3: Deaths per 1,000 live births [95% confidence interval] for early neonatal mortality, late neonatal mortality, post neonatal mortality, and child mortality by maternal education in 1993, 1999, 2006, 2016, and 2021

	No schooling	1st - 5th grade	6th - 8th grade	9th - 12th grade	Above 12th grade
**Early neonatal mortality**					
**1993**	37.5 [36.7-38.32]	30.95 [29.37-32.61]	26.75 [25.11-28.51]	19.51 [18.13-20.99]	19.75 [17.21-22.65]
**1999**	36.05 [35.22-36.89]	28.41 [26.98-29.91]	26.54 [24.95-28.22]	22.01 [20.68-23.42]	19.71 [17.34-22.4]
**2006**	34.24 [33.22-35.28]	34.16 [32.31-36.12]	23.45 [22.01-24.99]	21.56 [20.29-22.9]	14.28 [12.51-16.3]
**2016**	30.45 [29.97-30.94]	30.12 [29.36-30.9]	25.97 [25.27-26.68]	17.41 [16.96-17.87]	13.23 [12.68-13.8]
**2021**	27.52 [26.92-28.13]	23.6 [22.85-24.38]	23.07 [22.44-23.71]	17.17 [16.78-17.58]	11.29 [10.76-11.85]
**Late neonatal mortality**					
**1993**	17.18 [16.64-17.73]	9.12 [8.32-10]	9.27 [8.32-10.33]	7.74 [6.92-8.65]	2.75 [2.06-3.67]
**1999**	13.82 [13.32-14.34]	10.11 [9.24-11.05]	5.77 [5.05-6.59]	4.45 [3.91-5.05]	2.65 [1.89-3.7]
**2006**	10.17 [9.62-10.75]	9.62 [8.64-10.71]	8.01 [7.17-8.94]	4.89 [4.35-5.51]	1.31 [0.88-1.96]
**2016**	6.12 [5.91-6.35]	5.37 [5.03-5.72]	5.05 [4.73-5.39]	3.57 [3.37-3.78]	2.68 [2.32-3.08]
**2021**	5.66 [5.39-5.94]	5.83 [5.43-6.25]	3.88 [3.65-4.12]	3.25 [3.08-3.44]	2.44 [2.13-2.81]
**Post neonatal mortality**					
**1993**	37.02 [37.01-37.02]	24.69 [24.69-24.7]	21.35 [21.35-21.36]	13.48 [13.47-13.48]	6.42 [6.42-6.42]
**1999**	32.12 [32.12-32.13]	24.09 [24.09-24.09]	14.34 [14.34-14.34]	10.53 [10.53-10.53]	5.64 [5.63-5.64]
**2006**	25.15 [25.15-25.16]	17.61 [17.61-17.62]	13.89 [13.89-13.9]	9.09 [9.08-9.09]	5.04 [5.04-5.04]
**2016**	16.62 [16.62-16.62]	13.58 [13.57-13.58]	10.41 [10.41-10.42]	8.57 [8.57-8.57]	4.85 [4.85-4.85]
**2021**	15.85 [15.85-15.85]	13.93 [13.93-13.94]	10.87 [10.87-10.87]	8.38 [8.38-8.39]	5.94 [5.94-5.94]
**Child mortality**					
**1993**	42.61 [40.22-45]	20.94 [17.19-24.7]	16.55 [12.46-20.65]	7.64 [4.86-10.41]	1.92 [-0.8-4.65]
**1999**	39.64 [37.3-41.97]	20.32 [16.76-23.89]	11.82 [8.67-14.96]	7.16 [4.68-9.64]	4.33 [0.82-7.83]
**2006**	26.89 [24.47-29.31]	13.3 [10.03-16.57]	8.7 [5.86-11.54]	3.8 [2.3-5.29]	3.78 [0.59-6.96]
**2016**	15.1 [14.17-16.03]	10.74 [9.36-12.11]	6.86 [5.85-7.87]	4.39 [3.44-5.34]	2.6 [1.64-3.56]
**2021**	11.84 [10.76-12.92]	7.77 [6.57-8.97]	6.1 [5.06-7.13]	4.25 [3.61-4.9]	2.87 [1.91-3.83]

Supplementary figure 1: Standardized percent change for early neonatal, late neonatal, post neonatal, and child mortality by household wealth quintile and maternal education between 1993-1999, 1999-2006, 2006-2016, and 2016-2021

	Early neonatal mortality (first 7 days)				Late neonatal mortality (8-28 days)				Post neonatal mortality (29 days - 11 months)				Child mortality (12-59 months)			
	1993-1999	1999-2006	2006-2016	2016-2021	1993-1999	1999-2006	2006-2016	2016-2021	1993-1999	1999-2006	2006-2016	2016-2021	1993-1999	1999-2006	2006-2016	2016-2021
**Lowest**	-0.1	-0.5	-0.9	-3.3	-1.6	-4.6	-3.7	-4.2	-1.5	-5.6	-2.9	-1.9	0.0	-4.8	-5.0	-5.8
**Low**	-3.5	-0.5	-1.2	-2.9	-4.9	-2.2	-5.1	-1.1	-3.5	-1.6	-4.7	-2.7	-3.7	-5.6	-5.3	-6.2
**Middle**	-1.6	-1.4	-2.1	-3.7	-5.7	-1.5	-5.2	-2.9	-3.3	-3.0	-4.1	-1.0	-3.5	-6.0	-4.9	-4.6
**High**	1.3	-1.2	-3.0	-1.8	-4.7	-3.4	-4.0	-1.6	-5.7	-2.9	-3.5	1.6	-5.4	-6.6	-2.8	-4.0
**Highest**	1.1	-1.5	-3.8	-3.7	-5.2	-3.1	-0.8	-2.7	-4.5	-4.7	-2.7	-1.2	-2.4	-5.8	-4.1	2.6
**No schooling**	-0.6	-0.7	-1.1	-1.9	-3.3	-3.8	-4.0	-1.5	-2.2	-3.1	-3.4	-0.9	-1.2	-4.6	-4.4	-4.3
**1st to 5th grade**	-1.4	2.9	-1.2	-4.3	1.8	-0.7	-4.4	1.7	-0.4	-3.8	-2.3	0.5	-0.5	-4.9	-1.9	-5.5
**6th to 8th grade**	-0.1	-1.7	1.1	-2.2	-6.3	5.5	-3.7	-4.6	-5.5	-0.4	-2.5	0.9	-4.8	-3.8	-2.1	-2.2
**9th to 12th grade**	2.1	-0.3	-1.9	-0.3	-7.1	1.4	-2.7	-1.8	-3.6	-2.0	-0.6	-0.4	-1.0	-6.7	1.6	-0.6
**Above 12th grade**	0.0	-3.9	-0.7	-2.9	-0.6	-7.2	10.4	-1.7	-2.0	-1.5	-0.4	4.5	20.8	-1.8	-3.1	2.1

>0	<=0 & >-1.99	<=-2 & >-3.49	<=-3.5 & >-4.99	<= -5

Supplementary figure 2: Progress towards Sustainable Development Goal targets for ENMR, LNMR, PNMR, and CMR by household wealth quintile and maternal education as of 2021

	ENMR (target 7)			LNMR (target 5)			PNMR (target 8)			CMR (target 5)		
**Wealth quintile**	**2021 rate**	**Required SAC**	**Actual SAC**	**2021 rate**	**Required SAC**	**Actual SAC**	**2021 rate**	**Required SAC**	**Actual SAC**	**2021 rate**	**Required SAC**	**Actual SAC**
Lowest	27.8	-1.87	-1.08	5.3	-0.13	-0.29	14.8	-0.59	-0.30	11.6	-0.81	-0.94
Low	24.3	-1.53	-0.82	4.9	-	-0.06	11.7	-0.39	-0.37	7.3	-0.40	-0.66
Middle	18.9	-1.16	-0.85	3.8	-	-0.13	10.9	-0.25	-0.12	5.6	-0.16	-0.33
High	16.4	-0.78	-0.32	3.1	-	-0.05	9.0	-0.02	0.13	4.3	-	-0.22
Highest	9.4	-0.32	-0.42	2.4	-	-0.08	5.2	-	-0.06	3.2	-	0.07
**Maternal education**												
No schooling	27.5	-1.68	-0.59	5.7	-0.08	-0.09	15.90	-0.62	-0.15	11.8	-0.72	-0.65
1st to 5th grade	23.6	-1.65	-1.30	5.8	-0.03	0.09	13.90	-0.40	0.07	7.8	-0.41	-0.59
6th to 8th grade	23.1	-1.35	-0.58	3.9	-	-0.23	10.90	-0.17	0.09	6.1	-0.13	-0.15
9th to 12th grade	17.2	-0.74	-0.05	3.3	-	-0.06	8.40	-0.04	-0.04	4.3	-	-0.03
Above 12th grade	11.3	-0.44	-0.39	2.4	-	-0.05	5.90	-	0.22	2.9	-	0.05

Not on track | On track | Already met
