# Supplementary material for: Trends in socioeconomic inequality in mortality during childhood between 1993 and 2021 in India
Source: BMJ Glob Health. 2025 May 2;10(5):e016386. doi: 10.1136/bmjgh-2024-016386 (PMC12049899; doi:10.1136/bmjgh-2024-016386)
Supplement: online supplemental file 2 [file bmjgh-10-5-s002.docx]

# **Appendix S1 – Reflexivity Statement**

1. **How does this study address local research and policy priorities?**

Preventing child mortality remains a major research and policy priority for India’s government. Child mortality has fallen considerably throughout India over the past several decades. However, our study shows that India’s most socioeconomically marginalized children remain at the greatest risk for premature death. These findings can help local policy makes target interventions at these vulnerable populations.

1. **How were local researchers involved in study design?**

Of the six authors of this secondary data analysis and study, Dr. Soumya Swaminathan is considered a local researcher. Dr. Swaminathan is based in India and helped with the conceptualization and design of the study and provided critical revisions to the manuscript. Her involvement in these steps was essential given that she is an Indian paediatrician and her deep expertise in issues relating to public health in India. Furthermore, this was a secondary data analysis that did not require any field-level expertise or staff in India.

1. **How has funding been used to support the local research team?**

This study did not have dedicated funding for the research. The only funding support we have is for the article processing charge. Thus, participation of the Indian collaborator was purely out of policy and academic interest.

1. **How are research staff who conducted data collection acknowledged?**

This study is based on secondary data analysis and thus we did not rely on any local research staff.

1. **Do all members of the research partnership have access to study data?**

Yes, all members of the research partnership have access to the study data.

1. **How was data used to develop analytical skills within the partnership?**

Not applicable.

1. **How have research partners collaborated in interpreting study data?**

All the co-authors had access to the study data and participated in interpreting the results from our analysis.

1. **How were research partners supported to develop writing skills?**

Not applicable.

1. **How will research products be shared to address local needs?**

The only product of this research is an open-access peer-reviewed publication.

1. **How is the leadership, contribution and ownership of this work by LMIC researchers recognised within the authorship?**

Dr. Swaminathan is the lone LMIC researcher who participated in this study and she is part of the authorship team.

1. **How have early career researchers across the partnership been included within the authorship team?**

Dr. Anoop Jain and Mr. Akhil Kumar are both early career researchers and are the first and second authors, respectively.

1. **How has gender balance been addressed within the authorship?**

Two of the six authors on this study are women.

1. **How has the project contributed to training of LMIC researchers?**

There were no junior LMIC researchers involved in this study.

1. **How has the project contributed to improvements in local infrastructure?**

Not applicable.

1. **What safeguarding procedures were used to protect local study participants and researchers?**

The DHS data we used for this study are completely anonymized protecting the identities of all of the local study participants.
